# Amphotericin B Pharmacokinetics: Inter-strain Differences in Rats Following Intravenous Administration of the Most Commonly Marketed Formulations of the Drug

**DOI:** 10.5812/ijpr-134772

**Published:** 2023-03-23

**Authors:** Erfan Abdollahizad, Simin Dadashzadeh, Shima Bahri, Zahra Abbasian, Elham Rezaee

**Affiliations:** 1 Department of Pharmaceutics and Nanotechnology, School of Pharmacy, Shahid Beheshti University of Medical Sciences, Tehran, Iran; 2 Pharmaceutical Research Center, Shahid Beheshti University of Medical Sciences, Tehran, Iran; 3 Department of Medical Chemistry, School of Pharmacy, Shahid Beheshti University of Medical Sciences, Tehran, Iran

**Keywords:** Amphotericin B, Pharmacokinetics, Inter-Strain Differences, Wistar Rat, Sprague–Dawley Rat

## Abstract

**Background:**

Amphotericin B (AmB) is the first-line drug to treat invasive fungal infections. However, its delivery to the body and clinical use faces many challenges because of its poor solubility, poor pharmacokinetics, and severe nephrotoxicity.

**Objectives:**

Due to the necessity for designing safer and more effective nanocarriers for AmB and the importance of preclinical pharmacokinetic studies in evaluating these novel drug delivery systems, the present study was framed to explore the influence of rat strain on the pharmacokinetic profile of this drug.

**Methods:**

Twenty-four Wistar and Sprague–Dawley (SD) rats were intravenously injected with 1 mg/kg AmB as Fungizone or AmBisome, which are the two most commonly marketed formulations of the drug. Blood samples were collected before and at regular intervals up to 24 h after administration. Drug concentration was analyzed by a validated HPLC method, and pharmacokinetic parameters were determined by the non-compartmental method.

**Results:**

Irrespective of the type of formulation, the AUC_0-t_ and AUC_0-∞_ values were significantly higher (P < 0.001), and Cl as an important PK parameter was markedly lower (P < 0.001) in SD rats compared to the Wistar strain. For Fungizone, the mean Cl values in SD and Wistar rats were 206.90 and 462.95 mL/h/kg (P < 0.001), respectively. The apparent volume of distribution (V_ss_) was also lower in SD rats compared to Wistar; however, for AmBisome, the difference in V_ss_ was not statistically significant. Our further investigation suggested that the higher amount of total protein in the SD strain may justify the higher plasma concentrations and lower Cl and V_ss_ of amphotericin B in this strain compared to the Wistar strain.

**Conclusions:**

Overall, following intravenous administration of AmB, there were significant differences in the pharmacokinetic parameters of the drug between two rat strains for both formulations. The obtained data is important for correctly interpreting experimental data from different research groups.

## 1. Background

The prevalence of opportunistic fungal infections, which developed as secondary infections in Covid patients, considerably rose during the past few years with the emergence of the Covid-19 disease (1). As a result, medical professionals and researchers began to focus more on fungus infections and, naturally, antifungal therapeutic agents. Among the available antifungal medications, amphotericin B (AmB), due to its broad spectrum of activity and low resistance, is widely used and regarded as the gold standard (2). AmB interferes with the ergosterols of fungal cell membranes and has a fungicidal effect on various fungi, including opportunistic ones. However, its delivery to the body and clinical use faces many challenges due to its poor solubility, poor pharmacokinetics, and severe nephrotoxicity.

The intravenous (i.v.) formulation containing sodium deoxycholate surfactant, known as Fungizone, is the conventional formulation of AmB and is widely available. However, the liposomal form, known as AmBisome, is the preferred option in clinical use. The differences in pharmacokinetics (PK) of AmB following the administration of the two products are the root cause of this phenomenon. Liposomal form creates higher blood concentrations (more than ten times in the same prescribed dose compared to Fungizone) and maintains these concentrations during longer times (which is illustrated with a large area under the curve (AUC) in the plasma concentration-time curve) and facilitates antifungal effect. Additionally, a smaller apparent volume of distribution (V_ss_) of AmBisome renders it more difficult for the drug to get into tissues that are not infected, leading to less toxicity profile (3).

Despite the advantages of AmBisome, due to the generally lower physical stability of liposomes (4) and especially the complexity of the AmBisome manufacturing process, researchers are interested in developing newer formulations (5). The manufacturing process of this specific liposomal formulation is more complex than other liposomal products because it requires the complexation of AmB with distearoyl phosphatidyl-glycerol (DSPG) within the liposome bilayer (6). Moreover, its average particle size is less than 100 nm with low polydispersity; therefore, accurate and repeatable size reduction methods are required to obtain generic products comparable to the reference (5).

Scientists attempting to establish new drug delivery systems for AmB typically obtain the PK parameters of their developed system in rats, which are a suitable animal model for such studies, and compare their results with those reported for Fungizone and AmBisome. Looking at AmB PK data (Table 1) collected from previous reports (7-15), regardless of the type of formulation, AmBisome, or Fungizone, there are apparent differences between the results reported for the PK parameters of the drug. Due to the similarity of the medications and prescribed dosage, using different strains of rats in the previously reported studies may be an influential factor. Therefore, for a more detailed look, we examined and compared the PK of AmB in Wistar and Sprague-Dawley (SD) rats, two species that are often used in preclinical PK studies (16). To our knowledge, no similar study has been reported for AmB. Also, there are minimal reports dealing with the influence of the type of the drug delivery system on the magnitude of inter-strain differences in the PK profiles of drugs.

**Table 1.** Previously Reported Data for the PK Parameters of AmB in Rats After i.v. Administration

Medication	Dose (mg/kg)	Rat Strain	Sampling times (h)	AUC_0-t_ (last) (µg.h/mL)	AUC_0-∞_ (µg.h/mL)	V_ss_ (mL/kg)	Ref.
**Fungizone**	0.8	Wistar	0.5, 1, 2, 8, 12, 24, 48 and 72	-	2.3	3580 ^a^	(7)
**Fungizone**	0.8	SD	1, 2, 3, 4, 5, 6, 8, 10, 12, 24, 48 and 72	-	5	2924 ^b^	(8)
**Fungizone**	1	Wistar	0.5, 1, 2, 3, 5, 8, 12, 24, 48, and 72	4.4	-	3291 ^b^	(9)
**Fungizone**	1	Wistar	0.5, 1, 2, 4, 6, 24	4.8	-	4908 ^b^	(10)
**Fungizone**	1	SD	0.08, 0.17, 0.25, 0.5, 0.75, 1, 2, 4, 6, 8, 10 and 24	6	-	-	(11)
**Fungizone**	1	SD	0.08, 0.25, 0.5, 1, 2, 4, 8, 24	-	5	1170 ^a^	(12)
**AmBisome**	1	SD	0.25, 1, 3, 6, 12, or 24	43.1	-	207 ^a^	(13)
**AmBisome**	1	SD	0.08, 0.17, 0.25, 0.5, 0.75, 1, 2, 4, 6, 8, 10 and 24	35.5	-	-	(11)
**AmBisome**	1	Wistar	0.5, 1, 2, 4, 6, 24	64.3	-	167 ^b^	(10)
**AmBisome**	3	SD	0.5, 1, 3, 5, 8 and 24	-	290	145 ^b^	(14)
**AmBisome**	3	SD	0.5, 1, 2, 4, 8 and 24		219	233	(15)

^a^ Expressed as V_d_ in the original article or were calculated using the formula V_d_ = Dose / (AUC_0-∞_ × K) based on the reported data.

^b^ Calculated using the formula V_ss_ = Cl × MRT, based on the reported data in the related article.

## 2. Methods

### 2.1. Materials

Fungizone and AmBisome were supplied from Bristol-Myers Squibb (Princeton, USA) and Gilead Sciences (Cambridge, UK). Quantitative kits for total protein and albumin were acquired from Delta Darman Part (Tehran, Iran). HPLC-grade acetonitrile and methanol were supplied by Merck (Darmstadt, Germany).

### 2.2. Animal

All animal experiments for this study were conducted in accordance with the regulations of the Shahid Beheshti University of Medical Sciences animals Ethics Committee (registered ethics code of IR.SBMU.PHARMACY.REC.1398.277, Tehran, Iran). The male Wistar and SD rats were obtained from the Royan Institute (Tehran, Iran) and kept at a temperature range of 20 to 25°C, 50 percent humidity, a 12 h of light during 24 h, and unrestricted access to food. At the time of injection, all rats weighed 200 ± 20 g.

### 2.3. Study Design

Twenty-four Wistar and SD rats were randomly divided into four groups (n = 6 in each group) and administered either Fungizone or AmBisome. The commercial formulations were hydrated in accordance with their leaflet, diluted with dextrose 5% to a volume of 0.25 mL, and injected intravenously via the tail vein of rats at a dosage of 1 mg/kg of body weight. Blood samples (0.25 mL) were collected at 0.25, 0.5, 1, 2, 4, 6, 8, 10, 12, and 24 h following injection. In order to separate the plasma, they centrifuged for 5 min at 10000 rpm. The plasma samples were stored at -80°C until their analysis.

### 2.4. Drug Assay

The analysis of the drug in the plasma was conducted in line with the previous study (17) with minor modifications. Briefly, the technique of methanol precipitation was used in plasma sample preparation. 250 µL of methanol was added to 100 µL of plasma sample, followed by adding 50 µL of piroxicam solution as an internal standard (10 µg/mL in methanol). Then, they vortexed for 20 min and centrifuged at 10000 rpm for 10 min. The supernatant was transferred to a new micro-tube and dried at room temperature under a nitrogen gas flow. The dried sample was rehydrated with mobile phase, and then 100 µL of this solution, after centrifugation for 5 min at 10000 rpm, was injected into the HPLC.

As the mobile phase, a 47:53 v/v solution of acetonitrile and acetic acid (7.3%) with a pH adjustment of 3 was used. The analysis system used a K-1001 solvent delivery pump (Knauer, Germany) and a C-18 Perfectsil™ column (250 × 4.6 mm, with 5 µm particles, MZ-Analysentechnik GmbH, Germany), running at a flow rate of 1 mL/min at room temperature, and a Wellchrom K-2700 UV detector (MZ-Analysentechnik GmbH, Germany) set at 405 nm to measure the drug content. The minimum measurable concentration was 0.025 µg/mL, and accuracy, precision, and linearity of the analysis method were all attained throughout the measurement range (0.025 to 20 µg/mL).

### 2.5. Plasma Protein Assay

The total protein and albumin of the plasma sample of untreated rats were measured by using the total protein and albumin assay kits (Delta Darman Part, Tehran, Iran). Briefly, the total protein determination kit relied on biuret colorimetry. Existing proteins in an alkaline medium combine with copper ions to form azure complexes, and the intensity of the color is proportional to the amount of protein in the sample. The albumin kit is based on forming blue-green complexes of this protein with bromocresol green substance in acidic environments, the color intensity of which is proportional to the sample's albumin concentration. The UV-Visible spectrometer (Shimadzu, Japan) was used to determine the absorption of each sample, and the concentrations were calculated based on the absorption of standard solutions.

### 2.6. Pharmacokinetics and Statistical Analysis

Plasma PK parameters of AmB were calculated using the non-compartmental model by PKSolver software (an add-in program in Microsoft Excel) (18). The half-life (t_1/2_) was computed as 0.693 × K^-1^, where K is the elimination rate constant extracted from the slope of the last portion of the log-transformed plasma concentration-time curve. The linear trapezoidal rule was utilized to calculate the AUC from t = 0 to the last detected time (AUC_0-t_). The equation, AUC_0-∞_ = AUC_0-t_ + C_last_ /K, was used to calculate the extrapolated AUC from t = 0 to infinity, where C_last_ is the last measured concentration. Clearance (Cl), mean residence time (MRT), and volume of distribution at steady state (V_ss_) were calculated using the following equations (19):

Cl = Dose/ AUC_0-∞_

MRT = AUMC_0-∞_/AUC_0-∞_, where AUMC_0-∞_ (area under the first moment curve) is the area under the C * t plotted against t from time 0 to infinity.

V_ss_ = MRT × Cl

Data were presented at mean ± standard deviation (SD). For two-group comparisons, independent t-tests were used, and P < 0.05 was considered statistically significant. All statistical analyses were performed with SPSS version 16 (SPSS Inc., USA).

## 3. Results and Discussions

**Figure 1.**
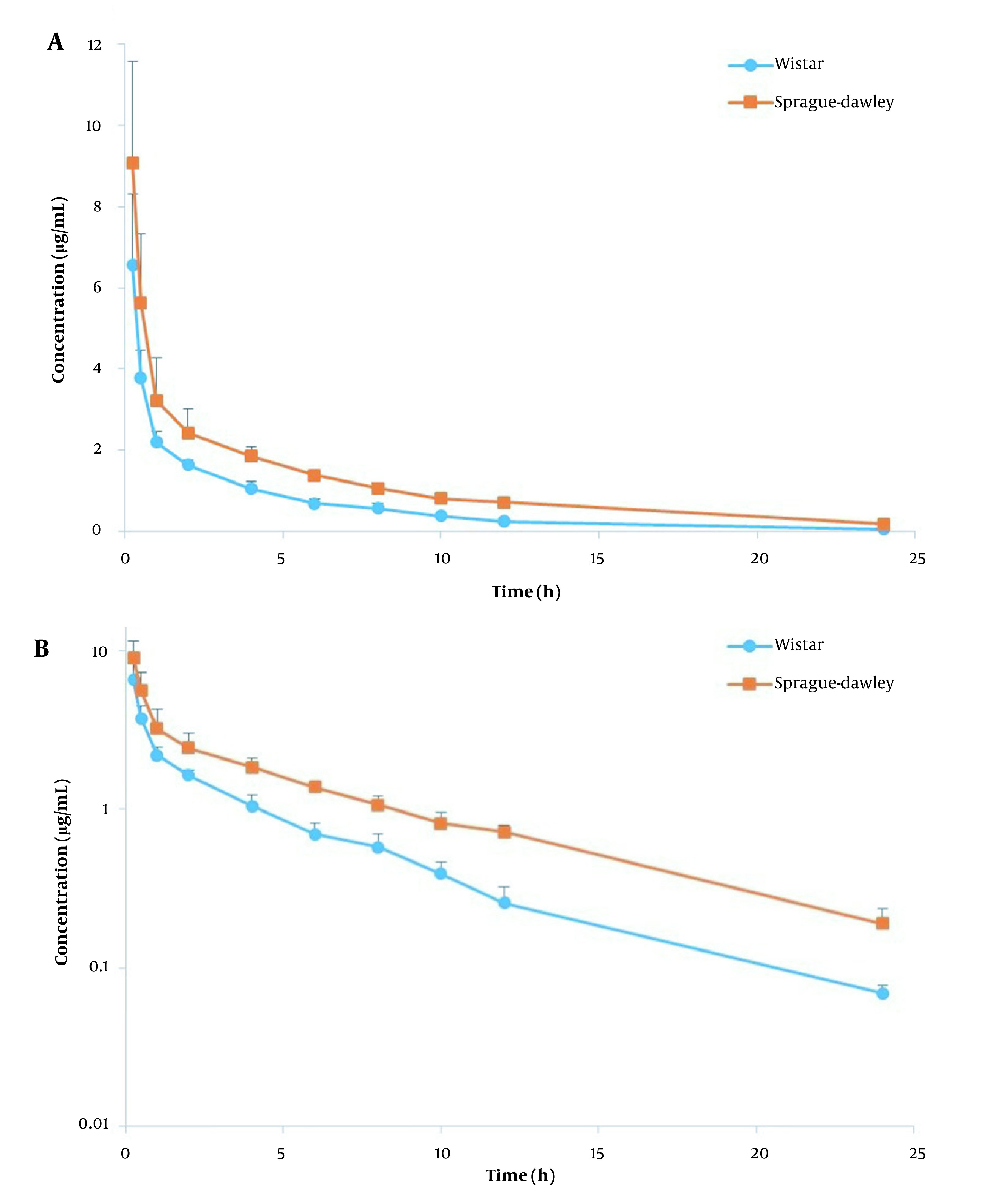
Plasma concentration-time profiles of amphotericin B (dose= 1 mg/kg) following i.v. administration of AmBisome in linear (A) and logarithmic (B) scale in Wistar and SD rats (n = 6, mean ± SD).

**Figure 2.**
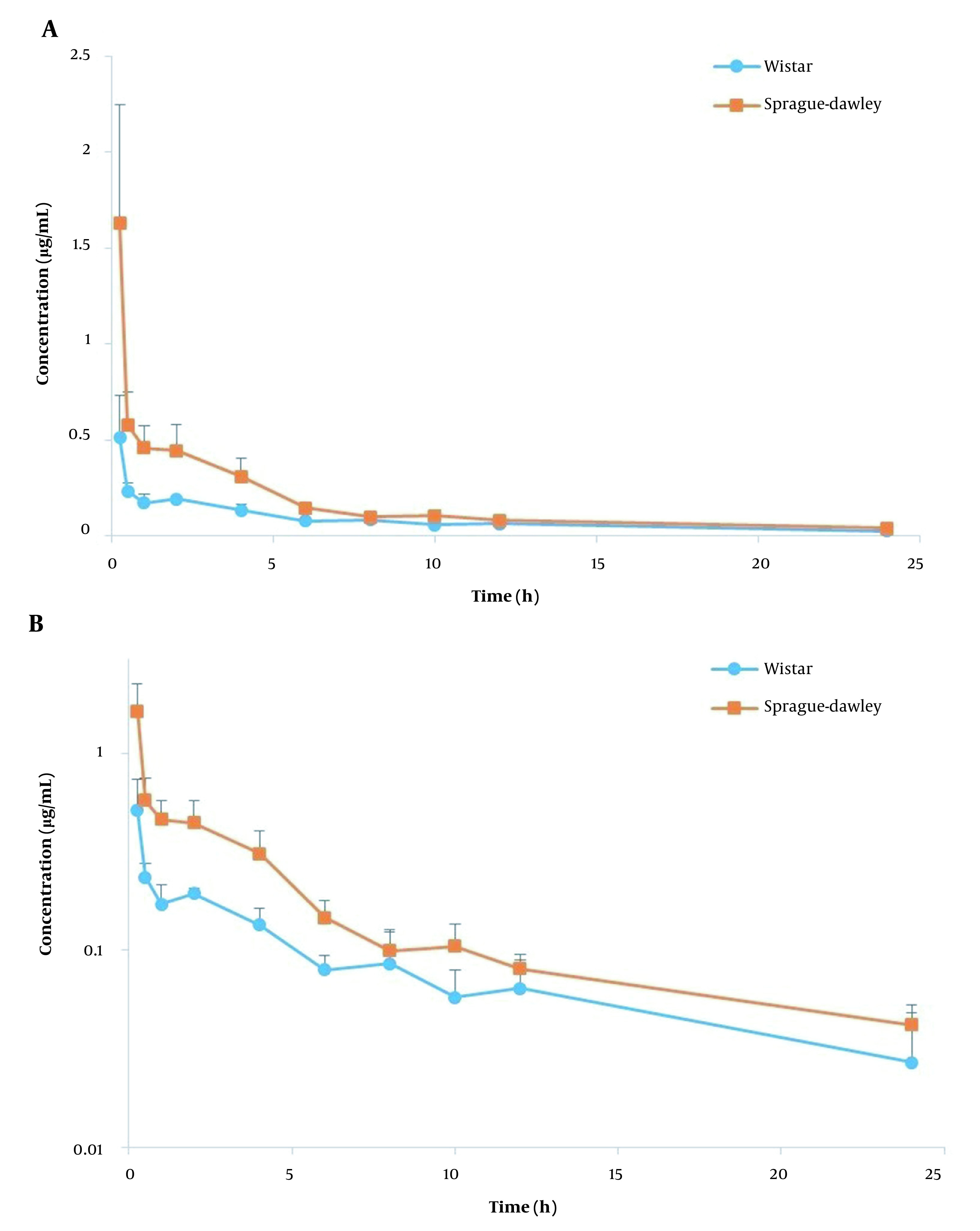
Plasma concentration-time profiles of amphotericin B (dose= 1 mg/kg) following i.v. administration of Fungizone in linear (A) and logarithmic (B) scale in Wistar and SD rats (n = 6, mean ± SD).

The plasma concentration-time profiles of AmB following i.v. injection of AmbBisome and Fungizone in Wistar and SD rats are shown in Figures 1 and 2, and a summary of PK parameters is illustrated in Table 2. The obtained PK parameters of Fungizone and AmBisome in each rat strain were consistent with those previously reported (7, 11, 12). In the comparison of the two formulations, as shown in Table 2, aside from the rat strain, the AUC_0-∞_ for AmBisome (ranging between 17.60 to 30.46 µg.h/mL) was 6 to 8 times higher than that of Fungizone (2.19 to 4.97 µg.h/mL) while the related V_ss_ and Cl were markedly smaller than that observed for the conventional formulation. For example, mean V_ss_ values were around 245.90 to 295.51 mL/kg for AmBisome versus 2091.60 to 3745.08 mL/kg for Fungizone (P < 0.001).

**Table 2.** PK Parameters of AmB in Wistar and SD Rats After i.v. Bolus Administration of Either Fungizone or AmBisome with the Drug Dose of 1 mg/kg (n = 6, mean ± SD).

Parameters	Fungizone			AmBisome		
	Wistar Rats	SD Rats	P-Value	Wistar Rats	SD Rats	P-Value
**AUC**_**0-24h**_ ** (µg.h/mL)**	2.05 ± 0.30	4.31 ± 0.30	< 0.001	17.22 ± 2.78	28.55 ± 4.42	< 0.001
**AUC**_**0-∞**_ ** (µg.h/mL)**	2.19 ± 0.29	4.97 ± 0.14	< 0.001	17.60 ± 2.74	30.46 ± 4.48	< 0.001
**Cl (mL/h/kg)**	462.95 ± 64.89	206.90 ± 15.65	< 0.001	58.14 ± 10.33	33.39 ± 4.47	< 0.01
**MRT (h)**	8.11 ± 2.11	10.01 ± 2.71	> 0.05 *	5.02 ± 0.57	7.33 ± 1.00	< 0.01
**t**_**1/2**_ ** (h)**	5.85 ± 1.29	11.96 ± 1.54	< 0.001	4.75 ± 1.01	6.72 ± 1.09	< 0.01
**V**_**ss**_ ** (mL/kg)**	3745.08 ± 982.84	2091.60 ± 604.69	< 0.01	295.51 ± 80.21	245.90 ± 53.45	> 0.05

The observed pharmacokinetic behavior for each product can be explained by their type of formulation and specific characteristics. Following i.v. administration of Fungizone, the first marketed formulation of AmB with deoxycholate, the drug molecules, leaves deoxycholate in the blood rapidly (20-22) and, therefore, can easily extravasate into tissues. In addition, they bind to the protein and/or cholesterol of tissue cells and thus have a large volume of distribution. Subsequently, the drug molecules distributed in the tissue cells return almost slowly to the plasma, creating a low concentration in the blood for an extended period and resulting in a prolonged elimination half-life (for Fungizone, the t_1/2_ mean value was about 12 h in SD rats).

The other formulation, AmBisome, is a liposomal formulation with an average particle size of around 100 nm, much bigger than small drug molecules. Therefore, these nanovesicles cannot easily distribute to various tissues of the body except those with a discontinuous and permeable endothelial network, and this feature results in a markedly smaller V_ss_ (21). Due to the same mechanism, initial plasma concentrations are elevated, and the AUC is larger than the conventional formulation. All these PK features of AmBisome improve the passive targeting of infected tissues (which have increased capillary vascular wall permeability) (3, 23). However, due to being a nanoparticle, it is finally removed from the blood by the mononuclear phagocyte system (24). It is worth mentioning that the lower plasma concentrations at initial times (shown in Figures 1 and 2) and a lower AUC for Fungizone, in comparison with AmBisome, limit its efficacy (21).

Regarding the influence of rat strain on the PK of AmB, which is the main purpose of the present study, it can be seen that irrespective of the type of formulation, the SD strain's AUC_0-t_ and AUC_0-∞_ were significantly higher (P < 0.001) and Cl as an important PK parameter was markedly lower (P < 0. 01) in SD rats compared to Wistar strain. V_ss_ was also lower in SD rats compared to Wistar; however, in the case of AmBisome, the difference was not statistically significant. For example, for Fungizone, the mean Cl values in SD and Wistar rats were 206.90 and 462.95 mL/h/kg (P < 0.001), and mean V_ss_ values were 2091 and 3745 mL/kg (P < 0.01), respectively.

In the previous studies performed on other drugs (16, 25-27), the PK differences between SD and Wistar rats have been typically attributed to metabolism variations, especially in the liver enzymes of these two rat strains. AmB has no known metabolism pathway and is dominantly excreted intact in feces (15, 28), so this explanation is less likely in the case of AmB. As mentioned before, AmB has a very high protein binding (29). Because a high plasma protein binding generally limits the distribution of xenobiotics from the plasma into the tissues where they could be eliminated, different degrees of protein binding in two strains of rats are likely involved in the observed differences.

**Figure 3.**
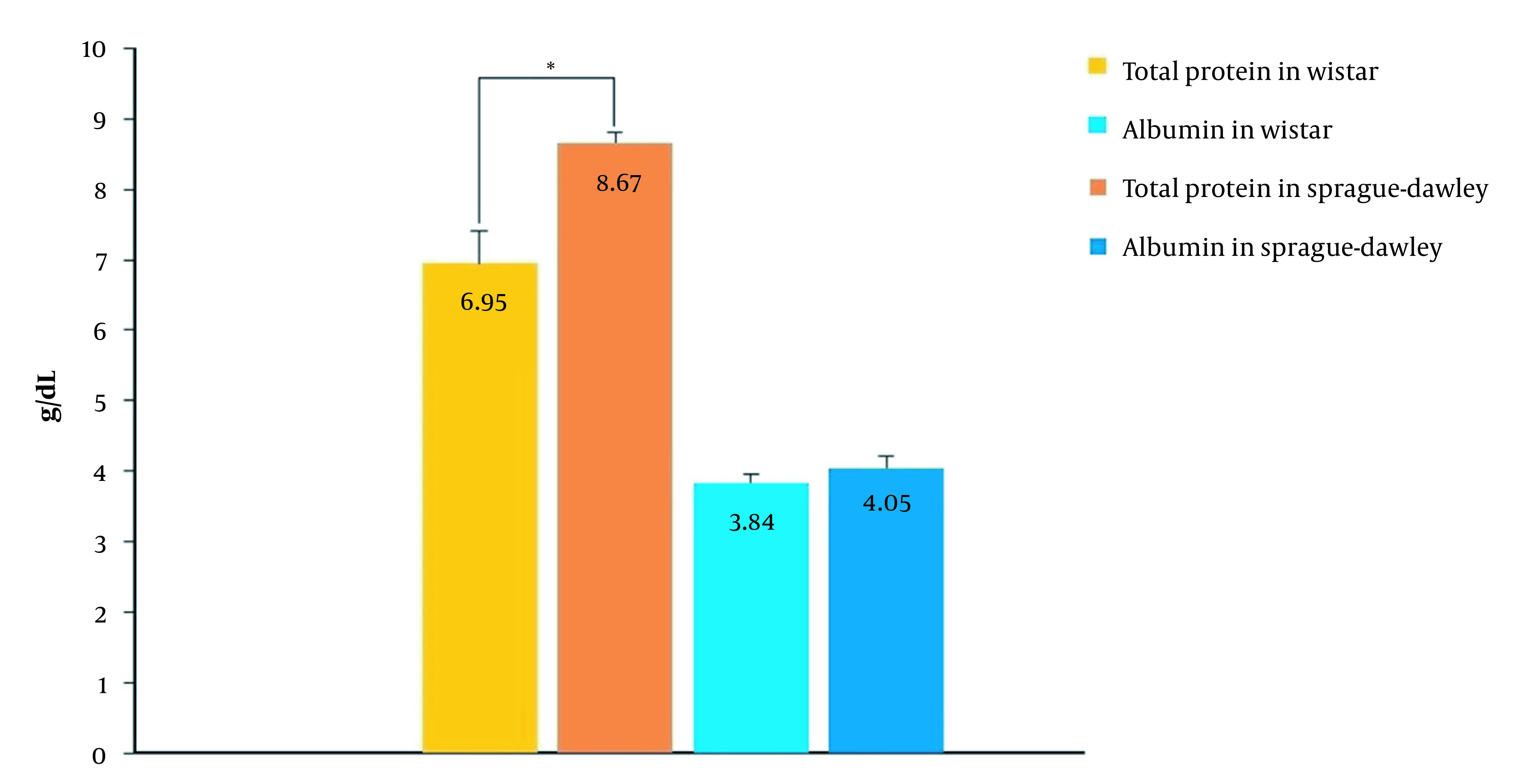
Total protein and albumin concentrations in plasma of Wistar and SD rats (n = 6, mean ± SD), * significant difference.

For this, we attempted to measure total protein and albumin levels in rat plasma. According to Figure 3, in the SD strain, the total protein level was about 25% more than the Wistar strain (P < 0.01), while the amounts of albumin were the same. Since AmB binds to albumin and non-albumin proteins (α1-acid glycoprotein and lipoproteins) (29), the differences in PK parameters of AmB between the two strains may be attributed to the different amounts of non-albumin proteins. This reason is especially likely for the conventional formulation.

For AmBisome, the drug molecules are predominantly encased in liposomes and are less available to bind to plasma proteins (2, 15). Therefore, it is expected that the inter-strain variation in the amount of plasma protein will have less effect on the PK of liposomal form compared to Fungizone. This explanation is supported by the results (Table 2) showing that for AmBisome, the magnitude of observed inter-strain differences in the PK parameters was smaller than for Fungizone.

Although in the case of liposomal form, the effect of plasma protein binding may be less, the inter-strain difference in the phagocytic power of the mononuclear phagocytes can also be an influencing factor. In a recent study by Guan et al. (30), the inter strains differences in the PK of liposomes in mice have been attributed to the variation in the number and phagocytic power of the phagocyte cells as well as to the variation in the content of opsonin and dysopsonin proteins (30).

## 4. Conclusions

This study demonstrated significant differences in the pharmacokinetic parameters of AmB between the two rat strains following i.v. administration of the two most common formulations, Fungizone and AmBisome. Some PK parameters showed a difference of more than twofold. Because AmB has no established metabolic pathway, the importance of this issue and investigating its causes increases. According to our findings, the SD strain plasma contained a higher total protein level, particularly non-albumin proteins, compared to Wistar rats. Therefore, different percentages of plasma protein binding may be one of the causes of observed between-strain differences in the PK parameters of AmB. Furthermore, the results show that the type of drug delivery system can be involved in the magnitude of observed inter-strain differences in the PK parameters of AmB. In the case of AmBisome, a liposomal formulation, it is necessary to conduct more research to determine whether differences in the number and phagocytic power of phagocytes in two strains can also affect the PK profiles. The obtained data is important for correctly interpreting experimental data from different research groups.

## Data Availability

The datasets generated and/or analyzed during the current study are available from the corresponding author upon reasonable request.
